# Muscle Fiber Conduction Velocity Correlates With the Age at Onset in Mild FSHD Cases

**DOI:** 10.3389/fphys.2021.686176

**Published:** 2021-06-17

**Authors:** Matteo Beretta-Piccoli, Massimo Negro, Luca Calanni, Angela Berardinelli, Gabriele Siciliano, Rossella Tupler, Emiliano Soldini, Corrado Cescon, Giuseppe D’Antona

**Affiliations:** ^1^Criams-Sport Medicine Centre Voghera, University of Pavia, Pavia, Italy; ^2^Rehabilitation Research Laboratory 2rLab, Department of Business Economics, Health and Social Care, University of Applied Sciences and Arts of Southern Switzerland, Manno, Switzerland; ^3^Child Neuropsychiatry, IRCCS Mondino Foundation, Pavia, Italy; ^4^Department of Clinical and Experimental Medicine, University of Pisa, Pisa, Italy; ^5^Department of Life Sciences, University of Modena and Reggio Emilia, Modena, Italy; ^6^Research Methodology Competence Centre, Department of Business Economics, Health and Social Care, University of Applied Sciences and Arts of Southern Switzerland, Manno, Switzerland; ^7^Department of Public Health, Experimental and Forensic Medicine, University of Pavia, Pavia, Italy

**Keywords:** neuromuscular disease, dystrophy, electromyography, fatigability, D4Z4, correlation, muscle fiber conduction velocity

## Abstract

A majority of patients with facioscapulohumeral muscular dystrophy (FSHD) report severe fatigue. The aim of this study was to explore whether fatigability during a performance task is related to the main clinical features of the disease in mildly affected patients. A total of 19 individuals with a molecular genetic-based diagnosis of FSHD (median D4Z4 deletion length of 27 kb) performed two isometric flexions of the dominant biceps brachii at 20% of their maximal voluntary contraction (MVC) for 2 min, and then at 60% MVC until exhaustion. Fatigability indices (average rectified value, mean frequency, conduction velocity, and fractal dimension) were extracted from the surface electromyogram (sEMG) signal, and their correlations with age, age at onset, disease duration, D4Z4 contraction length, perceived fatigability, and clinical disability score were analyzed. The conduction velocity during the low level contraction showed a significant negative correlation with the age at onset (*p* < 0.05). This finding suggest the assessment of conduction velocity at low isometric contraction intensities, as a potential useful tool to highlight differences in muscle involvement in FSHD patients.

## Introduction

Facioscapulohumeral dystrophy (FSHD) is the third most common hereditary myopathy worldwide, with an estimated prevalence of 1/15,000–1/20,000 (Mostacciuolo et al., 2009). The clinical heterogeneity of FSHD makes it difficult to predict the disease course; diagnosis depends on a combination of genetic and clinical features (Lemmers et al., 2012) and eventually, exclusion of other similar disorders. Diagnosis is facilitated by the fact that in most patients there is contraction in the DNA fragment on chromosome 4q35 containing the 3.3-kb D4Z4 repeat (Wijmenga et al., 1992; van Deutekom et al., 1993). The classical clinical presentation of FSHD includes asymmetric sequential face and shoulder girdle weakness followed by weakness in the foot extensor, abdominal, and hip girdle muscles caused by atrophy and fatty infiltration (Zampatti et al., 2019). The clinical features of FSHD were shown to be correlated with sex and age of onset (Zernov and Skoblov, 2019). Moreover, several genotype–phenotype correlation studies reported a rough inverse correlation between the number of D4Z4 repeats and the severity of FSHD (Lunt et al., 1995; Tawil et al., 1996; Ricci et al., 1999). However, more recent publications did not find satisfactory correlations, even using a large number of patients (Ruggiero et al., 2020; Salort-Campana et al., 2020; Vercelli et al., 2021).

In addition to muscle deterioration, severe fatigue is reported by a majority of FSHD patients (Hamel et al., 2019) and is an early manifestation of the disease that constrains the performance of common daily activities (Schipper et al., 2017). Despite its prevalence, fatigue in FSHD has not been adequately investigated and the extent to which performance and perceived fatigability limit cognitive and physical functions in patients is unknown (Kluger et al., 2013). Performance fatigability refers to a decline in objective measures of performance such as the production of maximal voluntary force, ability to provide an adequate signal to voluntarily activated muscle, and involuntary twitch response to stimulation (Enoka and Duchateau, 2016); it can be assessed by surface electromyography (sEMG) using spectral parameters, muscle fiber conduction velocity (CV), or nonlinear parameters (Rampichini et al., 2020). Fatigue during isometric constant force contractions is reflected by a decrease in CV mainly related to a change in intracellular pH (Komi and Tesch, 1979). A reduction in the fractal dimension (FD) of the sEMG signal has been linked to fatigability, aging, and disease (Goldberger et al., 2002; Arjunan and Kumar, 2013; Beretta-Piccoli et al., 2021), and is a useful index for assessing performance fatigability.

To date there have been no studies on the relationship between performance fatigability and genetic (e.g., D4Z4 allele size) and clinical (e.g., FSHD score) aspects of the disease. To address this issue, the present study investigated whether indices of performance fatigability extracted from the sEMG signal can predict the genetic and clinical features of patients with mild FSHD.

## Materials and Methods

### Participants

The study was part of a crowdfunded project (#Sport4therapy) carried out at the CRIAMS-Sport Medicine Centre Voghera supported by the University of Pavia that was aimed at identifying the correct sports therapy approach for patients affected by FSHD (Berardinelli and D’Antona, 2019). Data collection started in 2013 and was completed in 2019 (Beretta-Piccoli et al., 2021). Inclusion criteria were as follows: age of ≥ 16 years; clinical or genetic diagnosis of FSHD; and enrollment in the Italian National Registry for FSHD. Exclusion criteria were as follows wheelchair-bound at selection; use of corticosteroids; severe cardiac and respiratory dysfunction; and psychological or psychiatric disorders.

A diagnosis of FSHD was confirmed by DNA testing (Lemmers et al., 2012) at the University of Modena and Reggio Emilia (Modena, Italy). Disease severity was determined by FSHD clinical score (Lamperti et al., 2010), which ranges from 0 (no sign of functional impairment) to 15 (severe impairment of all tested muscle groups and wheelchair dependency). The scale is divided into six independent sections that assess the strength and functionality of facial (score 0–2), scapular girdle (score 0–3), upper limb (score 0–2), distal leg (score 0–2), pelvic girdle (score 0–5), and abdominal (score 0–1) muscles.

Patients were allocated to the four clinical categories of the Comprehensive Clinical Evaluation Form (Ricci et al., 2016), which classifies subjects presenting facial and scapular girdle muscle weakness (category A); subjects with muscle weakness limited to the scapular girdle or facial muscles (category B); asymptomatic or healthy subjects (category C); and subjects with a myopathic phenotype presenting clinical features not consistent with the canonical FSHD phenotype (category D).

Asymmetry of muscle involvement was clinically evaluated and patients with predominantly right or left side involvement were compared to determine whether side involvement was correlated with disease severity. Significant asymmetry (right-side predominance) in upper extremity muscle involvement was previously observed that was independent of handedness (Rijken et al., 2014). The participants’ characteristics are listed in Table 1.

**TABLE 1:** Clinical summary.

		*n*	Median	IQR
**Characteristics**				
Gender	Woman	10	–	–
	Man	9	–	–
Age (years)		–	33.50	31.25
Age at onset (years)		–	15.00	20.00
Disease duration (years)		–	10.00	23.00
FSHD categories	A	14	–	–
	B	3	–	–
	D	2	–	–
FSHD asymmetry	Right > Left	10	–	–
	Right = Left	6	–	–
	Right < Left	3	–	–
D4Z4 contraction (kb)/number of alleles		–	27.00	11.50
(11–19)/1–3		2		
(20–26)/4–5		7		
(27–31)/6		5		
(33–35)/7–8		3		
(36–41)/9–10		2		
D4Z4 contraction (kb)		–	27.00	11.50
Checklist individual strength^a^		–	26.00	15.00
Severity of FSHD (clinical score)		–	4.00	6.25
Scapular girdle involvement score			2.00	1.00

**^*a*^Variable with 3 missing values (n = 16). FSHD, facioscapulohumeral muscular dystrophy; IQR, interquartile range.**

All subjects provided written, informed consent to participate in this study, which was conducted according to the Declaration of Helsinki with approval from the local Ethics Committee of the University of Pisa.

### Fatigability Assessment

#### Perceived Fatigability

The fatigue severity subscale of the Checklist Individual Strength (CIS-fatigue) was used to assess trait levels of perceived fatigability before task performance. The CIS-fatigue consists of eight questions regarding fatigue experienced in the previous 2 weeks; each question was scored on a 7-point Likert scale (Vercoulen et al., 1994), with a total score ≥ 35 indicating severe fatigue (Vercoulen et al., 1996). The CIS-fatigue has good internal consistency (Cronbach’s α = 0.83–0.92), high discriminant validity, and high sensitivity to change in patients with FSHD (Kalkman et al., 2007).

#### Performance Fatigability

Performance fatigability was evaluated in FSHD patients as previously described (Beretta-Piccoli et al., 2017, 2020, 2021). Briefly, participants performed two maximal voluntary contractions (MVC) separated by 2 min rest, followed by a 20% MVC contraction lasting 2 min and a 60% MVC held until the force level decreased to < 90% of the target (endurance time). The two submaximal contractions were separated by 5 min rest. Because arm muscles show early disability in FSHD (Derry et al., 2012; Rijken et al., 2014; Tawil, 2018), the biceps brachii was selected for testing. Submaximal contractions were analyzed as their intensity is more representative of that associated with the performance of activities of daily living.

### EMG and Force Measurements

Myoelectric signals in the dominant biceps brachii were detected in monopolar configuration. Participants were seated on a height-adjustable chair with their arm positioned on an isometric ergometer (MUC1; OT-Bioelettronica, Turin, Italy) equipped with a load cell (Model TF022; CCT Transducers, Turin, Italy). The wrist was fastened to the ergometer, with the elbow flexed at 120°. A bidimensional array of 64 electrodes (3 mm diameter, 8 × 8 grid, 10 mm interelectrode distance) (model ELSCH064NM3; OT-Bioelettronica) was positioned on the biceps brachii as previously described (Beretta-Piccoli et al., 2020). The ground electrode was placed on the contralateral wrist.

Elbow torque was assessed using a torque meter operating linearly in the range of 0–1,000 Nm. The torque signal was amplified (MISO II; OT-Bioelettronica) and saved on a computer. EMG signals were amplified by a variable factor ranging from 2,000 to 5,000 (EMG-USB2+; OT-Bioelettronica), filtered with the hardware filter (10–500 Hz bandpass) followed by an offline Butterworth anticausal bandpass filter, and sampled together with the torque signal at 2,048 Hz using a 16-bit A/D converter with 5-V dynamic range and saved on a computer. The torque signal was displayed on a screen as real-time biofeedback.

### Signal Processing

The channels used for CV estimation were selected based on visual inspection of single differential signals along one of the array columns as previously described (Beretta-Piccoli et al., 2017); numbers typically ranged from 4 to 7 (Farina et al., 2004). CV was estimated using a multichannel algorithm (Farina and Merletti, 2003) on single differential signals using non-overlapping signal epochs of 1 s on the selected channels. Each of the selected signal epochs was used to estimate average rectified value (ARV), mean frequency of the power spectrum (MNF), and FD of the sEMG signal; these variables were averaged across all selected channels. ARV (a measure of the amplitude) and MNF (a parameter used to quantify changes in the spectral content of the sEMG signal based on the Fourier transform) were computed offline with numerical algorithms (Merletti et al., 1990). FD was estimated using the box-counting method (Gitter and Czerniecki, 1995). Performance fatigability was indirectly quantified as the slope of sEMG variables during the contractions.

### Statistical Analysis

Linear regression over time was applied to ARV, MNF, CV, and FD and the slopes were extracted and normalized with respect to their initial values. A Shapiro–Wilk test revealed that the variable distributions deviated from normality; consequently, the analyses were conducted using nonparametric statistical indicators. Relationships between continuous variables were investigated with Spearman’s correlation coefficient. Differences in fatigability parameters in relation to sex, FSHD categories, asymmetry of muscle involvement, and scapular girdle involvement score were assessed with the Mann–Whitney *U*-test. The statistical significance was set at α = 0.05. All statistical analyses were carried out with Stata/IC v16.0 (StataCorp, College Station, Texas, United States), and the results are reported as median and interquartile range (IQR).

## Results

### Clinical Features

Of the 19 patients, 14 belonged to category A and presented facial and scapular girdle muscle weakness (Ricci et al., 2016). Muscle weakness was more pronounced on the right side in 10 patients, 9 of whom were right-handed (Table 1); in 3 patients the weakness was greater on the left side and in 6, both sides showed equal weakness. The number of patients per D4Z4 contraction/number of repeats is shown in Table 1. The median FSHD clinical score was 4 (IQR = 6.25), indicating mild involvement of facial, scapular girdle, and upper limb muscles. The median scapular girdle involvement score was 2 (IQR = 1), suggesting moderate involvement with limitations in arm abduction (>45° but <180°) (Lamperti et al., 2010). Perceived fatigability as measured with the CIS-fatigue was mild (26 [IQR = 15]). The median MVC at 60° of isometric elbow flexion was 20.28 kg [IQR = 18.59].

### Correlation Analysis Between Fatigability and Clinical Variables

**FIGURE 1:**
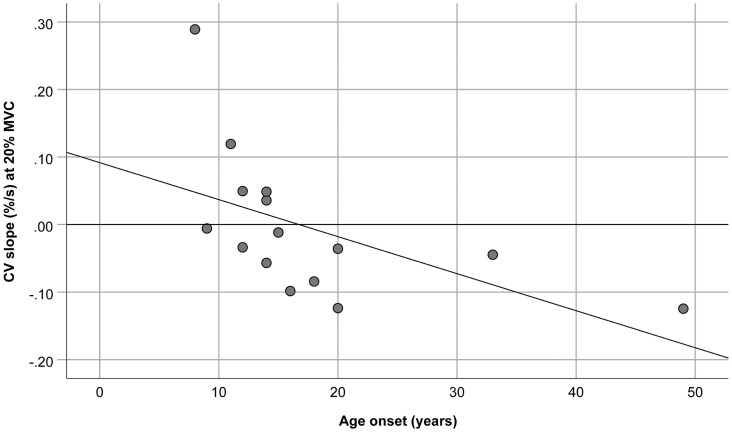
Relationship between the age at onset and muscle fiber conduction velocity (CV) slope during the 20% maximal voluntary contraction (MVC) of the biceps brachii. The slope was normalized with respect to the initial values. *R*^2^ = 0.29, *y* = 0.09–5.48E-3*x.

No significant correlation was observed between age, age at onset, disease duration, D4Z4 deletion length, CIS-fatigue score, clinical severity score, and rates of change in ARV, MNF, and FD. On the contrary, the rate of change of CV during the 20% MVC contraction significantly correlated with the age at onset (rho = −0.51, *p* < 0.05). When patients with borderline length of D4Z4 contraction (9–10 repeats) were excluded from the analysis, the significance dramatically increased (rho = −0.81; *p* < 0.001; Figure 1).

Within patients with a minimal D4Z4 contraction (1–5 alleles, *n* = 9), the following significant correlations were observed: (1) age with CV during the 60% MVC (rho = 0.86; *p* < 0.05); (2) age with MVC (rho = −0.77; *p* < 0.05); (3) disease duration with MVC (rho = −0.85; *p* < 0.01; Table 2).

**TABLE 2:** Spearman correlation coefficients between the continuous variables.

	% of the maximal voluntary isometric contraction	Age	Age at onset	Disease duration	D4Z4	CIS	FSHD_CS
D4Z4			0.2421	0.1697			
CIS^a^		0.0627	0.2818	0.0787	0.2045		
FSHD_CS		0.0840	0.0676	0.1142	–0.1952	0.0210	
ARV slope	20%	0.1537	0.1767	–0.1041	–0.0908	–0.0712	–0.0458
	60%	–0.0023	0.0572	0.1756	–0.0326	–0.2822	0.0371
MNF slope	20%	0.0603	–0.0176	–0.0071	0.1590	–0.1153	–0.3775
	60%	0.2945	0.3017	0.2118	0.0689	0.2172	–0.0396
CV slope^b^	20%	–0.1416	−0.5080*	–0.0333	0.0781	–0.2049	0.1287
	60%	0.1323	0.1587	0.1272	–0.0989	0.0086	0.1040
FD slope	20%	–0.0701	0.0149	–0.0485	0.0341	–0.0491	–0.2451
	60%	0.1461	0.2621	0.1959	–0.0114	0.2380	–0.0569
MVC^c^		–0.3366	–0.0232	–0.4491	0.2295	–0.1736	–0.3553
CV slope^d^	60%	0.857*					
MVC^d^		−0.766*		−0.854**			

*p < 0.05 and **p < 0.01.

*^*a*^Variables with 2 missing values (n = 17 instead of 19).*

*^*b*^Variables with 1 missing value (n = 18 instead of 19).*

*^*c*^Variables with 4 missing values (n = 15 instead of 19).*

*^*d*^Only patients with RU 1–5 were considered (n = 9 instead of 19).*

*ARV, averaged rectified value; MNF, mean frequency of the power spectrum; CV, conduction velocity; FD, fractal dimension; D4Z4, deletion length; CIS, checklist individual strength; CS, clinical score.*

### Differences in Fatigability According to Sex, FSHD Category, Asymmetry of Muscle Involvement, and Scapular Girdle Involvement Score

**FIGURE 2:**
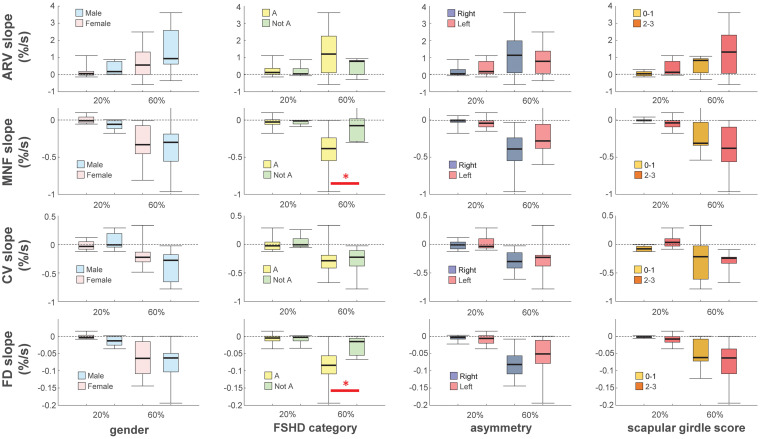
Box-and-whisker plots of slopes of average rectified value (ARV), mean power spectrum frequency (MNF), conduction velocity (CV) and fractal dimension (FD) during the 20 and 60% maximal voluntary contractions. Slopes were normalized with respect to their initial values. Asterisks denote statistical significance at ^∗^*p* < 0.05. Categories definition see text.

Because of the small sample size, in order to facilitate the assessment of differences in clinical variables we recoded FSHD category, asymmetry, and scapular girdle involvement score as binary variables [“A” and “Not A”; “Right > Left” and “Not right > Left”; and “normal abduction” (score 0–1) and “impaired abduction” (score 2–3), respectively]. There were no statistically significant differences in the performance fatigability parameters and maximal force according to sex, FSHD asymmetry, or scapular girdle involvement score. However, the median slope of MNF and FD during the 60% MVC contraction differed significantly between the A and Not A categories of FSHD (*p* = 0.026; Figure 2).

## Discussion

This study investigated (1) whether sEMG parameters that are indirect indices of performance fatigability are correlated with clinical variables of FSHD patients; and (2) the relationship between fatigability parameters and sex, FSHD category, asymmetry of muscle involvement, and scapular girdle involvement score. The results showed a significant correlation between muscle fiber CV and the age at onset. Moreover, a decrease in the slopes of MNF and FD during endurance contraction was observed in patients in category A compared to those in categories B and D (Not A).

### Relationship Between Fatigability and Clinical Variables

#### Age at Onset

In this study, we established that a late age at onset was associated with a more negative slope of muscle fiber CV, during the 20% MVC isometric elbow flexion, particularly in patients with 1–8 alleles. Patients with borderline D4Z4 contractions may have more mild phenotypes and older age at onset (Statland et al., 2015). In a previous publication on the same patients, we showed that this contraction level was not fatiguing, as the slope of CV was not different from zero (Beretta-Piccoli et al., 2021). Considering that at low level of muscle contraction graded recruitment prevails on rate coding to maintain the force (Milner-Brown et al., 1973), the observed negative correlation may mirror the overall lower deterioration of fast twitch fibers and/or fast to slow switch in biceps brachii when the disease clinical onset occurs later in life. Indeed, this finding identifies the assessment of CV at low % MVC isometric contraction as a potential useful tool to highlight differences in muscle involvement in FSHD patients.

#### D4Z4 Contraction and Clinical Score

No significant correlation was observed between D4Z4 contraction length and clinical variables or performance fatigability parameters. Although this has not been previously investigated, a negative correlation between muscle computed tomography grade and D4Z4 fragment size has been reported (Wang et al., 2012). Radiologic severity was shown to be unrelated to the size of the D4Z4 array (Olsen et al., 2006), but a correlation between the number of D4Z4 repeats and isokinetic peak torque of trunk extensors and flexors has been demonstrated (Esnault et al., 2018). Moreover, D4Z4 repeat size was shown to be correlated with total score for a manual muscle test (Sacconi et al., 2019). The observed variability in the correlation results may be related to the mean deletion length (Scionti et al., 2012). In fact, a borderline number of D4Z4 repeats (between 8 and 10) showed no association with the severity of clinical manifestations (Zernov and Skoblov, 2019).

Interestingly, within minimal D4Z4 contraction (1–5 repeats), the age of the patients showed significant correlations with the slope of the muscle fiber CV during the 60% MVC contraction, as well as with the MVC, suggesting that elderly patients are less prone to get fatigued, but at the same time generate lower levels of maximal force. Similarly, disease duration was negatively correlated to the exerted maximal force. In healthy subjects it is well known that a correlation between muscle fiber CV and age, characterized by a tendency of delay due to age, both during MVC and submaximal contractions, exists (Merletti et al., 1992; Hara et al., 1998; Bilodeau et al., 2001; Yamada et al., 2002; Bazzucchi et al., 2005; Mase et al., 2006). Differences between younger and older subjects become more apparent as motor units (MUs) recruitment becomes complete. In fact, the fast twitch, rapidly fatiguing fibers, being the later recruited MUs, are those more affected by the aging process. This is in agreement with the hypothesis that in older individuals, slow twitch fibers are predominant (D’Antona et al., 2003). Accordingly, reduction in maximal force in adult aging may be associated with alterations to the neuromuscular system, that affect the muscle fiber, the neuromuscular junction and the innervating motor neurons (Rice and Cunningham, 2002; Klass et al., 2007; Hunter et al., 2016). Data from the present investigation appear to corroborate the hypothesis that duration of the disease is a critical factor late deterioration of muscle force and fibers composition transition toward a slower phenotype and that this phenomenon appears to sum to the expected physiological change due to the aging process.

#### Perceived Fatigability

In line with previous findings in patients with FSHD (Schillings et al., 2007) or other neuromuscular disorders such as multiple sclerosis (e.g., Dodd et al., 2011; Wolkorte et al., 2015a; Severijns et al., 2016; Beretta-Piccoli et al., 2020), performance fatigability showed no correlation with clinical parameters. This may be because the patients in our study were not severely fatigued (CIS-fatigue < 35). Age and MVC values were shown to have positive and negative effects, respectively, on performance fatigability (Wolkorte et al., 2015b); these variables interact with each other and can interfere with the association between perceived and performance fatigability (Romani et al., 2004).

### Influence of Sex, FSHD Category, Asymmetry of Muscle Involvement, and Scapular Girdle Involvement Score on Fatigability

Performance fatigability parameters did not differ significantly according to most of the considered variables (sex, asymmetry, and scapular girdle involvement score). However, the slopes of MNF and FD during the endurance contraction differed between the two categories of FSHD (A and Not A), suggesting that patients with facial and scapular girdle muscle weakness experienced greater fatigue during isometric elbow flexion compared to the other patients. The two categories share pathophysiologic traits including impaired abduction of the upper limb with winged scapula and varying degrees of facial weakness (Ricci et al., 2016), which reflect a worsening phenotype (Vercelli et al., 2021). Importantly, the greater declines in the slopes of MNF (which is related to decreased muscle fiber CV and increased MU firing rate and synchronization (Bigland-Ritchie and Woods, 1984; Brody et al., 1991; Gabriel and Kamen, 2009) and FD (which is related to increased MU firing rate and synchronization Rampichini et al., 2020) suggest that the difference in fatigability between A and Not A is mainly due to central factors. This is supported by the results obtained for ARV and CV, which were altered during submaximal isometric contractions mostly as a result of peripheral factors (Dimitrova and Dimitrov, 2002, 2003; Dimitrov et al., 2006).

## Limitations

This study had some limitations. Firstly, the statistical power was low because of the small sample size. Therefore, the results should be considered as exploratory and confirmed in a larger cohort. Secondly, as the assessment of fatigability is task-dependent (Enoka, 1995), protocol specifications can affect the findings regarding the mechanisms underlying fatigue. Thirdly, we evaluated fatigability in the dominant biceps brachii only, which may not be representative of the disease as a whole and lastly, perceived fatigability is attributed to the whole body and does not necessarily correspond to fatigue in specific muscles.

## Conclusion

The length of D4Z4 contraction was not associated with disease severity or fatigability, indicating that unknown environmental factors have a significant impact on the genotype–phenotype interactions underlying clinical manifestations. However, trends in the fatigability parameters suggest more prominent fatigue in patients with a typical phenotype (category A) compared to other patient subsets.

## Data Availability Statement

The raw data supporting the conclusions of this article will be made available by the authors, without undue reservation.

## Ethics Statement

The studies involving human participants were reviewed and approved by University of Pisa. The patients/participants provided their written informed consent to participate in this study.

## Author Contributions

MB-P analyzed and interpreted the data and wrote the first and final drafts of the manuscript. MN supervised functional measurements. LC performed functional experiments. AB and GS contributed to medical examination of the patients and to writing and revision of the manuscript for intellectual content. RT participated in testing and medical tracking of the patients and contributed to writing and revision of the manuscript for intellectual content. ES performed statistical analyses. CC interpreted the data. GD’A developed the study concept, interpreted the data, and wrote the first and final drafts of the manuscript. All authors contributed to the article and approved the submitted version.

## Conflict of Interest

The authors declare that the research was conducted in the absence of any commercial or financial relationships that could be construed as a potential conflict of interest.
